# Comparison of different approaches for estimating age-specific alcohol-attributable mortality: The cases of France and Finland

**DOI:** 10.1371/journal.pone.0194478

**Published:** 2018-03-22

**Authors:** Sergi Trias-Llimós, Pekka Martikainen, Pia Mäkelä, Fanny Janssen

**Affiliations:** 1 Population Research Centre, Faculty of Spatial Sciences, University of Groningen, Groningen, The Netherlands; 2 Population Research Unit, Department of Social Research, University of Helsinki, Helsinki, Finland; 3 Centre for Health Equity Studies (CHESS), Stockholm University and Karolinska Institutet, Stockholm, Sweden; 4 Max Planck Institute for Demographic Research, Rostock, Germany; 5 Alcohol, Drugs and Addictions Unit, National Institute for Health and Welfare, Helsinki, Finland; 6 Netherlands Interdisciplinary Demographic Institute, The Hague, The Netherlands; Cliniques Universitaires Saint-Luc, BELGIUM

## Abstract

**Background:**

Accurate estimates of the impact of alcohol on overall and age-specific mortality are crucial for formulating health policies. However, different approaches to estimating alcohol-attributable mortality provide different results, and a detailed comparison of age-specific estimates is missing.

**Methods:**

Using data on cause of death, alcohol consumption, and relative risks of mortality at different consumption levels, we compare eight estimates of sex- and age-specific alcohol-attributable mortality in France (2010) and Finland (2013): five estimates using cause-of-death approaches (with one accounting for contributory causes), and three estimates using attributable fraction (AF) approaches.

**Results:**

AF-related approaches and the approach based on alcohol-related underlying and contributory causes of death provided estimates of alcohol-attributable mortality that were twice as high as the estimates found using underlying cause-of-death approaches in both countries and sexes. The differences across the methods were greatest among older age groups An inverse U-shape in age-specific alcohol-attributable mortality (peaking at around age 65) was observed for cause-of-death approaches, with this shape being more pronounced in Finland. AF-related approaches resulted in different estimates at older ages: i.e., mortality was found to increase with age in France; whereas in Finland mortality estimates depended on the underlying assumptions regarding the effects of alcohol consumption on cardiovascular mortality.

**Conclusions:**

While the most detailed approaches (i.e., the AF-related approach and the approach that includes underlying and contributory causes) are theoretically able to provide more accurate estimates of alcohol-attributable mortality, they–especially the AF approaches- depend heavily on data availability and quality. To enhance the reliability of alcohol-attributable mortality estimates, data quality for older age groups needs to be improved.

## Introduction

Excessive alcohol consumption is one of the major risk factors for morbidity and mortality, and the causal effects of the different dimensions of alcohol on various diseases and causes of death are relatively well established [1,2]. The effects of alcohol consumption on morbidity and mortality are more severe in Europe than elsewhere in the world [3,4] because of the high prevalence of drinking in Europe [5]. Estimates of alcohol-attributable mortality provide essential information about the harmful effects of alcohol at the population level. However, different estimation techniques yield different estimates. Moreover, the existing approaches seldom provide age-specific estimates, which can convey crucial information about the risk groups that should be targeted.

The estimation of alcohol-attributable mortality is a challenge for demographers, epidemiologists, and public health experts. Mortality statistics report data on ‘underlying causes of death’ (e.g., heart diseases, malignant neoplasm, cerebrovascular disease), and do not classify death according to the proximate behavioural cause of the occurrence of disease and injuries, such as alcohol consumption. While drinking alcohol is the only factor in some leading causes of death considered wholly attributable to alcohol (e.g., alcoholic liver cirrhosis or mental and behavioural disorders due to alcohol), alcohol can also be a contributing factor in the development of other diseases (e.g., ischaemic heart diseases or different types of cancer) and injuries [1].

In previous studies, a range of methods have been used to estimate alcohol-attributable mortality. These methods can be broadly divided into two groups that differ in their specifications. In the first group, only cause-specific mortality data are used. Studies using a selection of causes of death wholly attributable to alcohol have generally included the main underlying alcohol-attributable causes: i.e., mental and behavioural disorders due to the use of alcohol, alcoholic liver cirrhosis, accidental poisoning by alcohol, and other diseases that differ across studies [6–9]. These studies vary in the extent to which they take into account other causes of death when estimating alcohol-attributable mortality. For example, some studies include non-alcoholic liver cirrhosis [7,10,11] while others include causes of death that are partly attributable to alcohol [12]. Less frequently, both the contributing and the underlying causes of death are taken into account when estimating alcohol-attributable deaths (*underlying + contributory*) [13–16].

The second group of methods used to estimate alcohol-attributable mortality are methods based on attributable fractions (AF). By combining data on cause-of-death mortality, alcohol consumption, and dose-specific relative risks at different levels of drinking [17], these approaches take into account all deaths that are both wholly and partly attributable to alcohol. AF approaches have been widely used to estimate recent alcohol-attributable mortality not only in individual countries, such as in France [18,19]; but also worldwide using a harmonised methodology in the Comparative Risk Assessments at the Global Burden of Disease studies [3].

The choice of the estimation technique obviously affects the estimates obtained. Because AF approaches include mortality from causes of death that are both wholly and partly attributable to alcohol, studies that have used these approaches have provided information for selected countries and years on the relative importance of causes of death that are wholly and partly attributable to alcohol. These studies have shown that the estimates derived from AF approaches are at least twice as high as those derived from cause-of-death approaches, as the latter only include conditions that are wholly attributable to alcohol [18,19]. Marked differences in alcohol-attributable mortality estimates can also be observed when comparing approaches that include both the underlying and the contributing causes of death with approaches that include only the underlying causes that are wholly attributable to alcohol [14–16].

Many of the previous studies that provided alcohol-attributable mortality estimates distinguished between very broad age groups [17,19,20], and rarely between specific (five-year) age groups [13,21]. Age is, however, an essential determinant of alcohol-attributable mortality because of the age-specific differences in both current alcohol consumption (including drinking patterns) and the history of consumption over the individual life course [22]; and in the relationship between alcohol consumption and overall mortality [23]. Indeed, a study that distinguished between age groups noted the relative importance of including and dealing with older age groups in particular when estimating overall levels of alcohol-attributable mortality [20]. However, to the best of our knowledge, no previous study has directly compared the different methods used to estimate age-specific alcohol-attributable mortality.

We examine and compare for the first time the overall and the age-specific alcohol-attributable mortality estimates obtained by applying eight different estimation techniques to French and Finnish data. We chose to compare France and Finland because these two countries represent different drinking cultures with similar levels of current per capita consumption, but with very different levels of past per capita alcohol consumption. In France, per capita consumption of pure alcohol among adults (ages 15+) dropped from 21.1 litres in 1975 to 11.7 litres in 2010 [24]. Over the same period, per capita consumption of pure alcohol among adults in Finland increased from from 8.0 to 9.7 litres [24]; or from nine to 12 litres, if consumption that was not officially recorded is included [25]. Additionally, there are important differences in the drinking cultures of France and Finland, as the patterns of drinking and the levels of acceptance of drinking differ between the two countries [22,26]. In France, alcohol (mostly wine) has traditionally been consumed with meals. In Finland, by contrast, risky single-occasion drinking is still much more common than in France [27].

Recent studies for France have used AF approaches to estimate alcohol-related mortality, and have focused on causes of death more than on age-specific patterns. These studies provided estimates ranging from 20,255 (ages 15–75) to 36,500 (ages 15+) annual deaths (45–71 per 100,000) [18,19]. In Finland, a recent study that used the *underlying + contributory* approach as the benchmark method provided an estimate of around 2,500 (ages 25+) annual deaths (67 per 100,000) [13]. Because of the different methods used in these studies, levels of alcohol-attributable mortality in France and Finland cannot be readily compared.

## Materials and methods

### Data

We estimated sex-specific and five-year age-specific (ages 25–79) alcohol-attributable mortality in France (2010) and Finland (2013) using eight different definitions and methodologies that were previously used in the literature: namely, five specifications of the cause-of-death approach and three specifications of the AF approach (see further details under “methods”). For each method, alcohol-attributable mortality rates were estimated by five-year age groups by dividing death counts (estimates) by the corresponding population exposure. Due to the small sample size at younger ages, and in order to avoid potentially random variation in rates, we included deaths at ages 25 and older. We used the 75–79 age group as the oldest age group to ensure an accurate comparison across methods, which would not be possible with an open-ended age group.

For these estimates, mortality data by (underlying) causes of death and population exposures by age groups and sex were retrieved from the WHO Mortality Database [28]. In addition, detailed data and specifically tabulated data on underlying and contributory causes of death were obtained from Inserm CépiDc for France, and from the Statistics Finland for Finland.

For the attributable fractions (AF) approaches, we performed two estimations based on the methods and relative risks (RR) in Rehm and colleagues [17]. The alcohol consumption data used in these estimations were obtained from the Health and Social Protection Survey (*Enquête Santé et Protection Sociale*) [29] for France in 2010 and from the Health and Well-being for Residents Survey [30] for Finland in 2013. We also included the corresponding estimates from the Global Burden of Disease (GBD) Study 2013 [3,31].

All data was secondary and totally anonymized. No patients were involved in the design and implementation of the study.

### Methods

We used the following five cause-of-death approaches:

Underlying causes of death wholly attributable to alcohol (*underlying-wholly*): We considered 12 underlying causes of death (ICD-10) with an alcoholic aetiology: mental and behavioural disorders due to alcohol (F10), alcohol-related degeneration of the nervous system (G312), alcoholic polyneuropathy (G621), alcoholic myopathy (G721), alcoholic cardiomyopathy (I426), alcoholic gastritis (K292), alcoholic liver disease (K70), chronic pancreatitis with alcoholic aetiology (K860), fetal alcohol syndrome (Q860), accidental poisoning by alcohol (X45), intentional self-poisoning and exposure to alcohol (X65), and exposure and poisoning by alcohol with undetermined intent (Y15) [32].

Liver cirrhosis (*liver cirrhosis*): We considered cirrhosis-related underlying causes of death: alcoholic liver disease (K70), chronic hepatitis, not elsewhere classified (K73), and fibrosis and cirrhosis of liver and (K74) [10,11].

Short list of underlying causes of death wholly attributable to alcohol and liver cirrhosis (*main underlying*): We considered the three main diseases that are wholly attributable to alcohol, which accounted for >80% of the (underlying) causes of deaths that are wholly attributable to alcohol in both countries and for both sexes: behavioural disorders due to alcohol (F10), alcoholic liver disease (K70), and accidental poisoning by alcohol (X45). In addition, in line with previous studies (7), and in order to account for potential differences in coding practices in alcoholic liver cirrhosis [1], we included chronic hepatitis not elsewhere classified (K73) and fibrosis and cirrhosis of the liver (K74). Moreover, in contrast to the underlying approach, this specification has the advantage of only requiring three-digit ICD-10 codes.

The European Health for All Database definition (*HFA-DB*): The definition of alcohol-attributable mortality from the European Health for All Database [24] includes a selection of underlying causes of death that are wholly or partly attributable to alcohol consumption: cancer of the oesophagus (C15), cancer of the larynx (C32), alcohol dependence syndrome (F10), chronic liver disease and cirrhosis (K70, K73, K74, K76), and all external causes (V00-V99, W00-W99, X00-X99 and Y00-Y99) [33].

Underlying and contributory (*underlying + contributory*) causes of death: We included deaths for which alcohol consumption (ICD-10 codes: F10, G312, G4051, G621, G721, I426, K292, K70, K852, K860, O354, P043, Q860 and X45) was the underlying or a contributory cause [13,14].

We included three attributable fraction approaches in which estimates of deaths from conditions that are wholly attributable to alcohol are combined with estimates of deaths from conditions that are partly attributable to alcohol:

The conventional attributable-fraction approach (*AF-conventional*): Following Rehm and colleagues, we estimated the alcohol-attributable fractions (AAF) for the causes of death partly attributable to alcohol for each country, sex, and age using Levin’s formula [17]:

$${AAF}_{i}=\frac{\sum_{i=1}^{n}p_{i}({RR}_{i}-1)}{1+\sum_{i=1}^{n}p_{i}({RR}_{i}-1)}$$ (1)

Where *n* is the number of drinking categories, *p* is the proportion of drinkers, and *RR* are the relative risks of dying for each *i* alcohol consumption category. We defined four drinking categories: 0–19, 20–39, 40–59, and 60 or more grams of pure alcohol consumed per day. As is the case in most health surveys, the survey-based estimate of alcohol consumption underestimated total alcohol consumption based on sales in both countries. To adjust for unreported consumption, we followed previous work and modelled alcohol consumption using a Gamma distribution, shifting its parameters until the total matched the level of alcohol sales [34] that was obtained from the European Health for All Database [24].

The attributable-fraction approach, excluding (cardio)protective effects of alcohol (*AF (RR> = 1)*): This approach is identical to the conventional AF approach except that it excludes the (cardio)protective effects of alcohol on mortality, as these effects are disputed [35], and because none of the cause-of-death approaches includes the potential protective effects of alcohol on mortality.

Global Burden of Disease estimates (*AF-GBD*): the GBD 2013 study [3], which has often been cited in recent studies [36,37], estimated alcohol-attributable mortality by applying an alcohol-attributable fractions approach. GBD estimates differ from the other two AF methods in that they are based on different alcohol consumption estimates, the measure the risk of alcohol consumption on a continuous scale, and they use a narrower specification of causes of death [3,38] than is used in the conventional AF approach, which relies on detailed four-digit ICD-10 causes of death.

### Analyses & comparison

For all eight approaches, we estimated and compared the overall age-standardised (ages 25–79) and age-specific (five-year age groups) alcohol-attributable mortality rates (per 100,000) for each method, sex, and country.

In order to provide further insight into the differences between the more detailed methods (AF approaches and the *underlying + contributory* approach), we disentangled different forms of age-specific alcohol-attributable mortality into groups of underlying causes of death: cancers (ICD-10 codes: C00-D48), cardiovascular diseases (I00-I99), digestive disorders (K00-K99), external causes (S00-Y98), mental diseases (F00-F99), and other causes. For reasons of data availability, we could not perform such an analysis for the *underlying + contributory* method in Finland.

## Results

At ages 25–79, the age-standardised alcohol-attributable mortality rates among men ranged from 24.7 to 129.9 deaths per 100,000 in France, and from 49.2 to 165.5 deaths in Finland (Table 1). Among women, the alcohol-attributable mortality rates ranged from 8.4 to 42.9 deaths in France, and from 16.7 to 54.2 deaths in Finland. The lowest estimates were obtained by selecting *liver cirrhosis* only, while the highest estimates were obtained by applying the *HFA-DB* approach. The most detailed approaches (the AF-related approaches and the *underlying + contributory* approach) resulted in estimates of alcohol-attributable mortality that were around twice as high as the estimates provided by the *underlying-wholly*, *liver cirrhosis*, and *main underlying* approaches in both countries and for both sexes. The *underlying + contributory* approach resulted in higher estimates than the AF-related approaches in Finland, but not in France. The alcohol-attributable mortality rates were generally higher in Finland than in France, except when the *AF-GBD* approach (men only) and the *AF-conventional* approach (women only) were applied. Regardless of the method used, alcohol-attributable mortality was found to be higher among men than among women.

**Table 1:** Total alcohol-attributable deaths and age-standardised alcohol-attributable mortality rates (per 100,000) in France (2010) and Finland (2013) for men and women, ages 25–79.

		France (2010)		Finland (2013)	
		Men	Women	Men	Women
Approach	Method	deaths (rate)	deaths (rate)	deaths (rate)	deaths (rate)
Cause-of-death (CoD)	Underlying-wholly	5,875 (30,2)	1,746 (8,4)	1,423 (79,4)	393 (21,5)
	Liver cirrhosis	4,806 (24,7)	1,748 (8,4)	8,81 (49,2)	304 (16,7)
	Main underlying	7,202 (37,0)	2,329 (11,2)	1,265 (70,6)	416 (22,8)
	HFA-DB	25,302 (129,9)	8,901 (42,9)	3,104 (173,2)	990 (54,2)
	Underlying + Contributory	12,581 (64,6)	3,221 (15,5)	2,966 (165,5)	665 (36,4)
AF	AF-conventional	17,147 (88,1)	5,644 (27,2)	1,869 (104,3)	381 (20,9)
	AF (RR> = 1)	18,720 (96,1)	6,546 (31,6)	2,314 (129,2)	600 (32,9)
	AF-GBD	19,034 (97,7)	5,770 (27,8)	1,655 (92,4)	569 (31,2)

**Fig 1:**
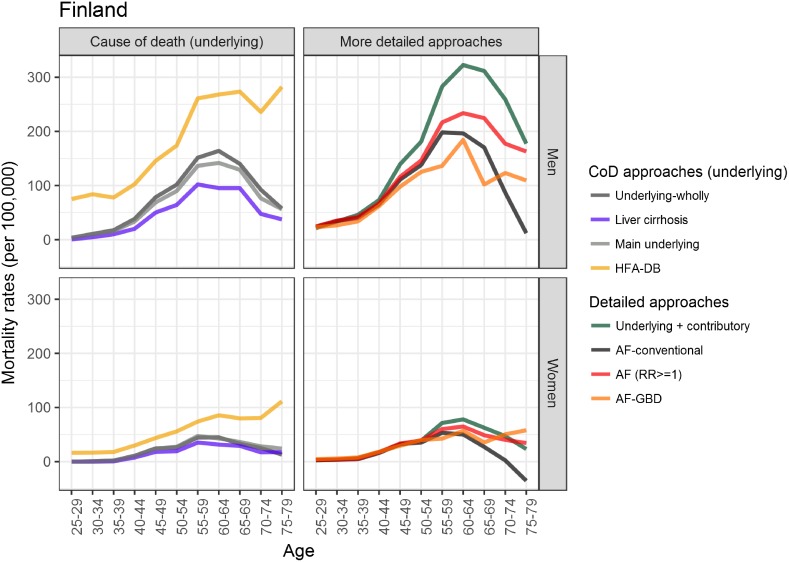
Age-specific alcohol-attributable mortality rates in Finland (2013) for men and women, ages 25–79.

**Fig 2:**
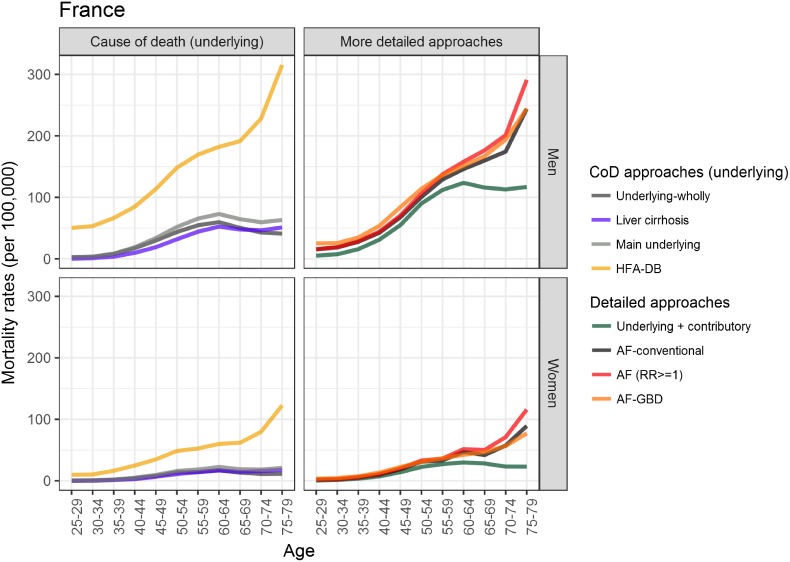
Age-specific alcohol-attributable mortality rates in France (2010) for men and women, ages 25–79.

With the exception of the estimates from the *HFA-DB* method, the age-specific estimates from the methods that use cause-of-death data exhibited similar patterns, regardless of gender and country: an increase until around age 65 and a decline at older ages, which is depicted as a reverse U-shape (Figs 1 and 2). The decline at older ages seems to be less pronounced in France than in Finland, however (S1 Fig). Among the older age groups, the AF approaches resulted in a wide range of estimates for Finland. In addition, differences between the countries were observed: e.g., there was a clear increase in alcohol-attributable mortality rates with age in France, but not in Finland (except among Finnish women when the *AF-GBD* approach was applied). When the estimates derived from the AF-related approaches were compared with those from the *underlying + contributory* method, differences in the results from the *AF* method and the other AF-related approaches were observed, but only among Finns aged 50+. In France, the *underlying + contributory* method estimated lower mortality than the AF-related methods for both men and women, and especially for the older age groups.

**Fig 3:**
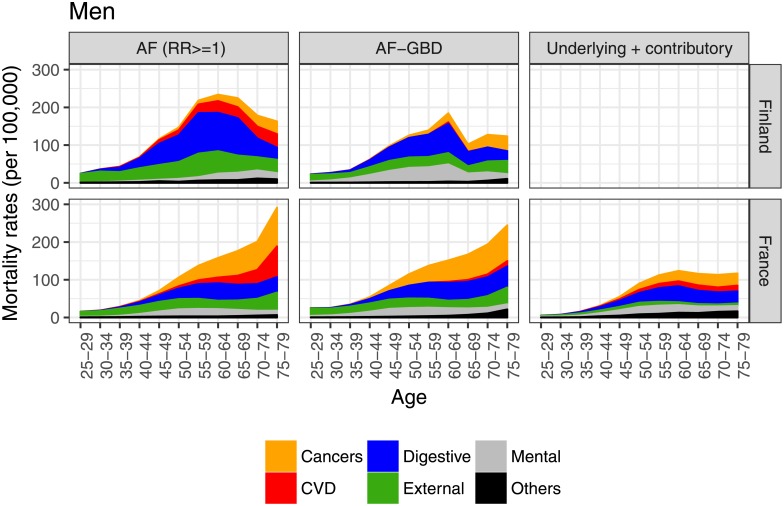
Cause-specific alcohol-attributable mortality rates in France (2010) and Finland (2013) for men, ages 25–79, by detailed method^ab^. ^a.^ Preventive mortality (negative numbers) was excluded in the AF-GBD method (only for CVD diseases and among men): for France, the minimum rates at ages 30–59 were equal to -1.23/100,000; for Finland, the rates at ages 55–59, 70–74, and 75–79 were -2.80, -4.20, and -13.94 per 100,000, respectively. ^b.^ Cause-specific death rates for the underlying + contributory approach for Finland were not available.

**Fig 4:**
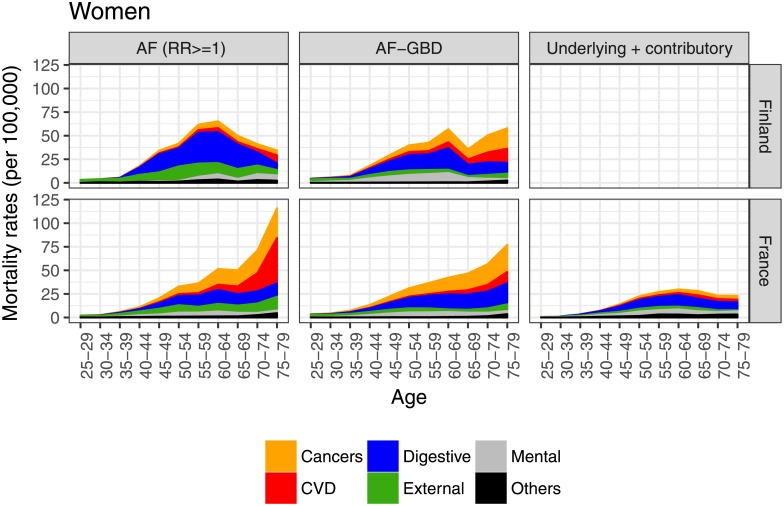
Cause-specific alcohol-attributable mortality rates in France (2010) and Finland (2013) for women, ages 25–79, by detailed method^ab^. ^a^. Preventive mortality (negative numbers) was excluded in the *AF-GBD* method (only for CVD diseases and among men): for France, the minimum rates at ages 30–59 were equal to -1.23/100,000; for Finland, the rates at ages 55–59, 70–74, and 75–79 were -2.80, -4.20, and -13.94 per 100,000, respectively. ^b^. Cause-specific death rates for the *underlying + contributory* approach for Finland were not available.

The cause-specific results of the AF-related approaches showed that the composition of the causes of death was relatively similar for both countries and for both men and women until around age 50 (Figs 3 and 4). Among the older age groups, the differences increased, especially in terms of cardiovascular and cancer mortality. These increases in rates of cancer and cardiovascular alcohol-related mortality with age seem to have been less pronounced in Finland. There were important differences between the estimates of cardiovascular mortality using AF-related approaches, with the estimates from the *AF (RR> = 1)* method being higher than the estimates from the *AF-GBD* method. When comparing AF-related methods to the *underlying + contributory* method in France, we observed that the higher estimates from AF-related methods among younger age groups were mostly due to external causes. Among older age groups, the higher alcohol-attributable mortality estimates provided by AF-related approaches were mainly due to higher estimates of cancer and cardiovascular alcohol-attributable mortality, which also increased more with age than in the estimates generated by the *underlying + contributory* approach. Finally, and across all age groups, estimates of mortality from external causes were lower when the *underlying + contributory* method was used than when AF-related methods were applied.

## Discussion

This study compared the overall and the age-specific alcohol-attributable mortality rates provided by eight different estimation techniques in France (2010) and Finland (2013). The overall mortality rates varied widely depending on the method applied: the methods that used additional data on either contributory causes or alcohol consumption and information on the association with mortality provided estimates that were around twice as high as the estimates generated by methods based solely on underlying causes of death that are wholly attributable to alcohol. The differences in the estimates provided by the various methods were especially large at older ages. Cause-of-death approaches generally estimated an inverse U-shaped age pattern, with a decline in alcohol-attributable mortality rates beyond age 65. Approaches that included alcohol consumption data and information on the association between alcohol consumption and mortality resulted in a similar inverse U-shaped pattern in Finland, but in increasing mortality with age in France due to high levels of alcohol-related cancer and cardiovascular mortality. Overall, however, higher levels of alcohol-attributable mortality were observed in Finland than in France.

### Interpretation of the results

The substantial differences in the estimates of alcohol-attributable mortality provided by the eight methods studied are in line with the differences that were previously found between the underlying approach and the AF approach in France [19], and between the underlying approach and the *underlying + contributory* approach in Finland [14]. The differences in the estimates can be traced back to both to the general approach used (cause-of-death approaches versus AF approaches) and to the details and the data associated with the particular methods. In general, more detailed approaches (AF approaches and *underlying + contributory*) theoretically provide more accurate estimates, as they account for the impact of alcohol on diseases and causes of death that are not fully attributable to alcohol, as well as on those that are wholly attributable to alcohol. These higher estimates should therefore be considered more reliable estimates of the total impact of alcohol on mortality than the estimates that account for a selection of underlying causes of death only. On the other hand, the estimates that account for underlying causes of death only can be considered estimates of the minimum burden of alcohol on mortality. In addition, causes of death wholly attributable to alcohol may not be fully recorded in death certificates due to stigma associated with alcohol-related health problems, which has been shown to be the case at least for alcoholic liver cirrhosis and alcoholic cardiomyopathy [39,40]. One of the approaches using underlying causes of death included all underlying liver cirrhosis (*main underlying*), and showed a similar age-specific pattern as the other underlying cause-of-death approaches (except the *HFA-DB*). Therefore, it seems that the underreporting of causes wholly attributable to alcohol by the physicians writing death certificates only has a rather minor impact on the age-specific alcohol-attributable mortality pattern. The estimates provided by the *HFA-DB* approach, which were the largest in both countries and for both men and women, were the most likely to be overestimates of the total burden of alcohol on mortality because the approach defined all external deaths as alcohol-related. By contrast, the *AF-conventional* approach defined only 25% of external deaths in Finland and 12% of external deaths in France as alcohol-related.

The differences in the alcohol-attributable mortality rates found using the various methods were largest at older ages, with the differences being greater between the different AF approaches than between the different underlying cause-of-death approaches (with the exception of the HFA-DB approach) (S2 Fig). The differences between the results of the AF approaches can be at least partly explained by data demands and methodological details. The AF approaches require not only data on cause-specific mortality; but also data on alcohol consumption and on the association between alcohol consumption and mortality that do not come from the same sources, and that require additional assumptions. The limitations and the potential biases of AF-related methods have been described elsewhere by several authors, including Rehm, Marmet, and Rey; see, e.g. [17,20,41]. The estimates for older age groups in particular are more sensitive to the data and assumptions used [20]. In our application, at least four specific factors that may cause bias should be taken into account. First, alcohol consumption estimates at older ages might be less reliable because of the smaller sample sizes for these age groups in surveys. Indeed, for France we had a sample size of less than 310 for each age group and sex at ages 65 and older (S1 Table). Second, because of the lack of questions in health surveys about lifetime alcohol consumption patterns, estimates of current drinking behaviour, especially at older ages, may not provide a full picture of the respondents’ lifetime exposure to alcohol. Third, efforts to estimate cause-specific mortality could be hampered by competing causes at older ages [42]. Furthermore, the estimates of the association between alcohol consumption and mortality are often derived from adult (and not older) populations [43], even though it is not clear that the risks are identical over age groups. Finally, the differences we observed between the *AF-conventional* approach and the *AF-GBD* approach seem to be mostly related to differences in alcohol consumption data, and less on the choice of a categorical measurement scale in the *conventional-AF* methods vs. a continuous scale in the *AF-GBD* method. Indeed, the observed differences between these two methods were rather tiny, except for older Finns. In the calculation of the *AF-conventional* estimates we used alcohol consumption data from the Health and Well-being for Residents Survey, which had a rather large sample size, also at old ages (S1 Table), and is considered a good source of consumption data, whereas the *AF-GBD* used forecasted data using different sources as input.

Our observation that alcohol-related mortality estimates at older ages vary greatly for Finland depending on the AF-based approach used, but are relatively similar for France, seems to point to another important factor driving old-age estimates. This difference between the countries may be related to the relative importance of causes of death for which alcohol has large cardioprotective effects in combination with the prevalence of moderate drinkers, to whom these cardioprotective effects tend to apply. The details of our analyses showed that the impact of alcoholic ischaemic heart disease on total alcohol-attributable mortality was much larger in Finland than in France. In general, we found that ischaemic heart disease mortality was three to four times higher in Finland than in France, which has also been documented elsewhere [44]. Ischaemic heart disease is not only the most prevalent cardiovascular cause of death; it is also the cause with the greatest cardioprotective effect [17]. This could explain the large differences observed between the results from the *AF-conventional* and *AF (RR> = 1)* approaches for Finland. In addition, the larger shares of moderate older drinkers in Finland than in France (S2 Table) contributed to the differences in the results from the AF-related methods in Finland. Clearly, the estimates of the (cardioprotective) effects of alcohol consumption on mortality have a notable impact on the estimates of alcohol-related mortality at older ages (when mortality itself is higher), especially in populations with higher mortality from causes of death for which cardioprotective effects may be significant, and with large shares of moderate drinkers.

In Finland, the estimates at older ages differed substantially depending on the AF approach used. However, we observed a reverse U-shaped age pattern irrespective of the method applied (except for the HFA-DB method). A reverse U-shaped pattern was also observed in France when the causes-of-death approach was used, albeit with a less pronounced decline in alcohol-related mortality at old ages; but not when the AF approaches were applied, as the estimates of these approaches showed an exponential increase in mortality rates at older ages. Because this exponential increase was observed only for the estimates from the AF approaches in France, and because of the above-mentioned data issues with the use of AF approaches at older ages, particularly given the small survey sample available, we are sceptical about this age pattern. Although the other approaches, including the detailed *underlying + contributory* approach, estimated an inverse U-shaped pattern for both countries and sexes, we cannot be fully certain that this is indeed the correct age pattern in France. Although the *underlying + contributory* approach seems to have been less affected by data quality issues than the AF approaches, underestimation could have occurred. Only in cases in which autopsies were carried out we can be relatively confident about the cause of death. Because autopsies are carried out less often when the cause of death is from a chronic disease than when it is accidental, underestimation is more likely to occur at old ages [14]. In addition, cancer-related alcohol-attributable mortality is likely to be underestimated because alcohol consumption and other risk factors that may have increased the likelihood of cancer are often not recorded in the death certificate. As a result, the decline in alcohol-attributable mortality rates with age that we observed at older ages may have been overestimated, and may have concealed a slightly different pattern than the observed inverse U-shaped age pattern.

In addition to these methodological and data differences between France and Finland, the overall patterns and the age patterns of alcohol-related mortality vary by the national context. Overall, higher levels of alcohol-attributable mortality were observed in Finland than in France, except when the *AF-GBD* method was applied to men and the *AF-conventional* method was applied to women. Examining the age patterns more in detail, we observed that when cause-of-death approaches were used, the differences between France and Finland were especially large among the middle-aged groups; and that when AF approaches were applied, alcohol-related mortality at older ages was higher in France than in Finland (S1 Fig). Alcohol-attributable mortality declined abruptly with age in Finland, but more moderately or not at all in France. Although the comparison between countries was hampered by data quality issues (see above), and by differences in how the physicians in each country have been trained to record causes of death [45], the observed differences can be at least partly linked to national differences in the patterns of drinking. Alcohol consumption is slightly higher in France than in Finland [24], but drinking patterns are riskier in Finland than in France [4,27]. These risky drinking patterns likely explain the higher rates observed in Finland, especially among young and middle-aged individuals. Additionally, the older generations in France and Finland were exposed to very different country-specific cultural practices related to alcohol use in their younger adulthood [26], which likely shaped their drinking behaviour over the life course. For example, older Finns have grown up in a dry society, and most have remained light drinkers or abstainers through their lives [22]; whereas older French people have grown up in a rather permissive alcohol culture, and overall levels of alcohol consumption have been much higher among this generation than among their younger counterparts [46]. The different patterns of lifetime exposure to alcohol use among the older generations in the two countries explain the more pronounced decline observed (using all methods) in alcohol-attributable mortality rates with age in Finland than in France, and illustrate that the actual age pattern depends on the context as well.

### Reflections on the choice of method for assessing alcohol-related mortality

The more detailed approaches used to estimate alcohol-attributable mortality (*AF-conventional*, *AF-GBD*, and *underlying + contributory*) theoretically provide more accurate estimates of the overall level of alcohol-related mortality. However, these more detailed approaches all require detailed data of high quality. The AF-related approaches require data drawn from three main sources, and rely on (often problematic) data on alcohol prevalence and on disease incidence at various levels of alcohol consumption. We argue that if highly accurate cause-specific (underlying and contributory) mortality data are available, the use of the *underlying + contributory* approach is recommended, as the estimates from this approach are less affected by additional assumptions. The underlying + contributory approach has commonly been applied in Finland [13,14] and Sweden [47], which have high-quality, cause-specific mortality data [48,49]; and has only recently been applied to other countries [15,16]. The application of the *contributory* approach in our study resulted in estimates that were lower than the estimates from AF-related approaches for France, especially among the younger and the older age groups; but not for Finland. However, adding the contributory cause of death to estimate alcohol-attributable mortality increased the rates in France by 75% for men and by 40% for women relative to the estimates from the method based on underlying causes only.

In addition to assessing the pros and cons of various methods for estimating alcohol-attributable mortality in one point of time it is also interesting to discuss the main strengths and limitations of the different methods to examine trends over time and in cross-national comparisons. Despite its high degree of accuracy of the *underlying + contributory* approach, it is impossible to use this approach to assess time trends in most European countries or to conduct cross-national comparisons on a large scale because contributory cause-of-death data are scarcely available for many countries and periods of time. Of the methods that use underlying causes of death, the *underlying* and the *main underlying* methods are, by definition, the most accurate, as the age patterns found when using these methods are similar to those observed when using the *underlying + contributory* method. Obviously, the overall levels found when using methods that take into account only the underlying causes of death are underestimates. However, these methods estimated an age pattern that was similar to the pattern found when using the *underlying + contributory* approach, but are easier to use because they do not require additional data or a set of assumptions. Because of potential country differences in the classification of liver disease mortality (alcoholic or other) [1], we recommend using the *main underlying* method, at least for comparative studies across countries and over time, although country-specific coding practices and changes therein over time should be carefully considered in the comparison. Generally, approaches that take into account underlying causes of death that are wholly attributable to alcohol follow the trends in per capita alcohol consumption over time (with a certain lag time) [6,50]. Thus, the estimates from these approaches may be seen as indicating the presence of other chronic conditions partly attributable to alcohol [9], especially when we examine time trends, and not merely levels.

## Conclusions

To the best of our knowledge, this is the first study that has compared underlying cause-of-death methods and methods based on more detailed data to estimate overall and age-specific alcohol-attributable mortality in different European countries. Our comparison of the overall and the age-specific alcohol-attributable mortality estimates from the application of eight different estimation techniques to French and Finnish data showed that the methods that relied on more detailed data (on either contributory causes of death or alcohol prevalence, and on their association with mortality) were more likely than other methods to provide accurate estimates of overall alcohol-attributable mortality levels; but are also dependent on the level of detail and the quality of these data. In the approaches that require information on the association between alcohol consumption and mortality, and in particular in the AF approaches, these data quality issues could explain the different age patterns we observed. A clear inverse U-shaped age pattern in alcohol-attributable mortality rates was found for Finland; but not for France, where the age-specific alcohol-attributable mortality pattern was different, in part because the older population in France had a long history of drinking. To enhance our knowledge about the impact of alcohol on mortality, and in order to further improve overall estimates of alcohol-attributable mortality, particular attention should be paid to the older age groups.

## Supporting information

S1 Table Valid cases by age, sex, and survey in France (2010) and Finland (2013). (DOCX)

S2 Table Adjusted alcohol prevalence in France (2010) and Finland (2013), by age and sex. (DOCX)

S1 Fig Comparison of age-specific alcohol-attributable mortality rates between France (2010) and Finland (2013) for men and women, ages 25–79. (DOCX)

S2 Fig Age-specific alcohol-attributable mortality rates in France (2010) and Finland (2013) for men and women, ages 25–79 (logarithmic scale). AF method for Finnish women aged 75–79 is excluded from the plot for visualization reasons as the rate is negative (-35.4).
(DOCX)

S1 Data Detailed datasets and data availability. (XLSX)
