# Supplementary material for: Comparison of different approaches for estimating age-specific alcohol-attributable mortality: The cases of France and Finland
Source: PLoS One. 2018 Mar 22;13(3):e0194478. doi: 10.1371/journal.pone.0194478 (PMC5864025; doi:10.1371/journal.pone.0194478)
Supplement: S1 Table — (DOCX) [file pone.0194478.s001.docx]

**Additional file 1**

**S1 Table.** Valid cases by age, sex, and survey in France (2010) and Finland (2013)

|  | France (ESPS) | | Finland (ATH) | |
| --- | --- | --- | --- | --- |
| Age | Men | Women | Men | Women |
| 25-29 | 366 | 408 | 1,029 | 1,525 |
| 30-34 | 353 | 487 | 1,134 | 1,528 |
| 35-39 | 487 | 546 | 1,178 | 1,529 |
| 40-44 | 491 | 547 | 1,167 | 1,512 |
| 45-49 | 533 | 607 | 1,386 | 1,873 |
| 50-54 | 486 | 597 | 1,652 | 2,182 |
| 55-59 | 485 | 503 | 1,992 | 2,364 |
| 60-64 | 432 | 467 | 2,325 | 2,763 |
| 65-69 | 308 | 294 | 2,300 | 2,544 |
| 70-74 | 256 | 285 | 1,431 | 1,794 |
| 75-79 | 248 | 267 | 2,132 | 2,767 |
