# Supplementary material for: Comparison of different approaches for estimating age-specific alcohol-attributable mortality: The cases of France and Finland
Source: PLoS One. 2018 Mar 22;13(3):e0194478. doi: 10.1371/journal.pone.0194478 (PMC5864025; doi:10.1371/journal.pone.0194478)
Supplement: S2 Table — (DOCX) [file pone.0194478.s002.docx]

**Additional file 2**

**S2 Table.** Adjusted alcohol prevalence in France (2010) and Finland (2013), by age and sex

| **Finland** |  |  |  |  |  |  |  |  |  |  |  |
| --- | --- | --- | --- | --- | --- | --- | --- | --- | --- | --- | --- |
|  |  |  |  |  |  |  |  |  |  |  |  |
| Men |  |  |  |  |  |  |  |  |  |  |  |
|  | 25-29 | 30-34 | 35-39 | 40-44 | 45-49 | 50-54 | 55-59 | 60-64 | 65-69 | 70-74 | 75-79 |
| 0 gr/day | 10% | 8% | 8% | 10% | 11% | 11% | 13% | 15% | 19% | 25% | 31% |
| 0-20 gr/day | 46% | 47% | 43% | 39% | 37% | 35% | 38% | 38% | 38% | 42% | 41% |
| 20-40 gr/day | 23% | 23% | 23% | 22% | 22% | 21% | 21% | 21% | 20% | 19% | 17% |
| 40-60 gr/day | 11% | 11% | 12% | 13% | 13% | 13% | 12% | 12% | 11% | 8% | 7% |
| 60+ gr/day | 10% | 11% | 14% | 16% | 17% | 20% | 16% | 15% | 12% | 6% | 4% |
|  |  |  |  |  |  |  |  |  |  |  |  |
| Women |  |  |  |  |  |  |  |  |  |  |  |
|  | 25-29 | 30-34 | 35-39 | 40-44 | 45-49 | 50-54 | 55-59 | 60-64 | 65-69 | 70-74 | 75-79 |
| 0 gr/day | 13% | 13% | 15% | 14% | 13% | 15% | 17% | 23% | 30% | 39% | 56% |
| 0-20 gr/day | 65% | 71% | 66% | 63% | 58% | 57% | 55% | 54% | 53% | 52% | 39% |
| 20-40 gr/day | 17% | 14% | 15% | 18% | 20% | 19% | 19% | 17% | 13% | 8% | 5% |
| 40-60 gr/day | 4% | 2% | 3% | 4% | 6% | 6% | 6% | 5% | 3% | 1% | 0% |
| 60+ gr/day | 1% | 0% | 1% | 1% | 3% | 3% | 3% | 2% | 1% | 0% | 0% |
|  |  |  |  |  |  |  |  |  |  |  |  |
| **France** |  |  |  |  |  |  |  |  |  |  |  |
|  |  |  |  |  |  |  |  |  |  |  |  |
| Men |  |  |  |  |  |  |  |  |  |  |  |
|  | 25-29 | 30-34 | 35-39 | 40-44 | 45-49 | 50-54 | 55-59 | 60-64 | 65-69 | 70-74 | 75-79 |
| 0 gr/day | 16% | 18% | 14% | 13% | 17% | 10% | 10% | 12% | 11% | 16% | 16% |
| 0-20 gr/day | 49% | 43% | 41% | 40% | 34% | 36% | 33% | 32% | 30% | 30% | 28% |
| 20-40 gr/day | 18% | 18% | 19% | 19% | 17% | 19% | 18% | 18% | 17% | 17% | 17% |
| 40-60 gr/day | 9% | 9% | 11% | 11% | 11% | 12% | 12% | 12% | 12% | 11% | 12% |
| 60+ gr/day | 9% | 12% | 16% | 17% | 21% | 23% | 26% | 27% | 30% | 26% | 28% |
|  |  |  |  |  |  |  |  |  |  |  |  |
| Women |  |  |  |  |  |  |  |  |  |  |  |
|  | 25-29 | 30-34 | 35-39 | 40-44 | 45-49 | 50-54 | 55-59 | 60-64 | 65-69 | 70-74 | 75-79 |
| 0 gr/day | 34% | 38% | 27% | 30% | 24% | 24% | 30% | 21% | 28% | 35% | 39% |
| 0-20 gr/day | 49% | 45% | 51% | 48% | 45% | 43% | 42% | 41% | 40% | 31% | 28% |
| 20-40 gr/day | 10% | 10% | 12% | 12% | 15% | 15% | 14% | 16% | 14% | 13% | 12% |
| 40-60 gr/day | 4% | 4% | 5% | 5% | 7% | 8% | 7% | 9% | 7% | 8% | 7% |
| 60+ gr/day | 3% | 3% | 4% | 5% | 8% | 10% | 8% | 13% | 10% | 12% | 13% |
