# Supplementary material for: Comparison of different approaches for estimating age-specific alcohol-attributable mortality: The cases of France and Finland
Source: PLoS One. 2018 Mar 22;13(3):e0194478. doi: 10.1371/journal.pone.0194478 (PMC5864025; doi:10.1371/journal.pone.0194478)

**Additional file 3**

**S1 Figure.** Comparison of age-specific alcohol-attributable mortality rates between France (2010) and Finland (2013) for men and women, ages 25-79
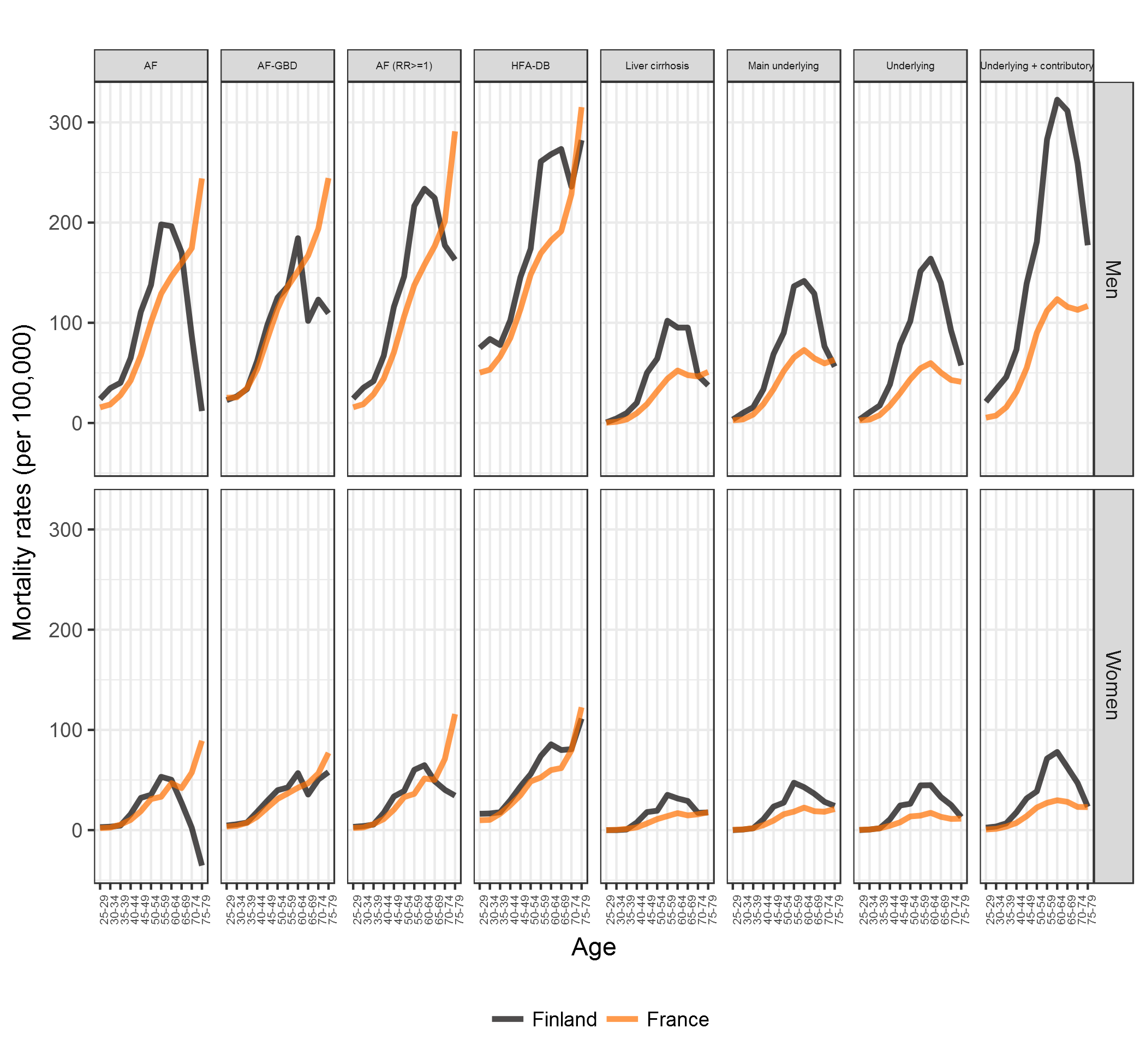

Supplement: S1 Fig — (DOCX) [file pone.0194478.s003.docx]
