# Supplementary material for: Comparison of different approaches for estimating age-specific alcohol-attributable mortality: The cases of France and Finland
Source: PLoS One. 2018 Mar 22;13(3):e0194478. doi: 10.1371/journal.pone.0194478 (PMC5864025; doi:10.1371/journal.pone.0194478)
Supplement: S2 Fig — AF method for Finnish women aged 75–79 is excluded from the plot for visualization reasons as the rate is negative (-35.4). (DOCX) [file pone.0194478.s004.docx]

**Additional file 4**

**S2 Figure.** Age-specific alcohol-attributable mortality rates in France (2010) and Finland (2013) for men and women, ages 25-79 (logarithmic scale)
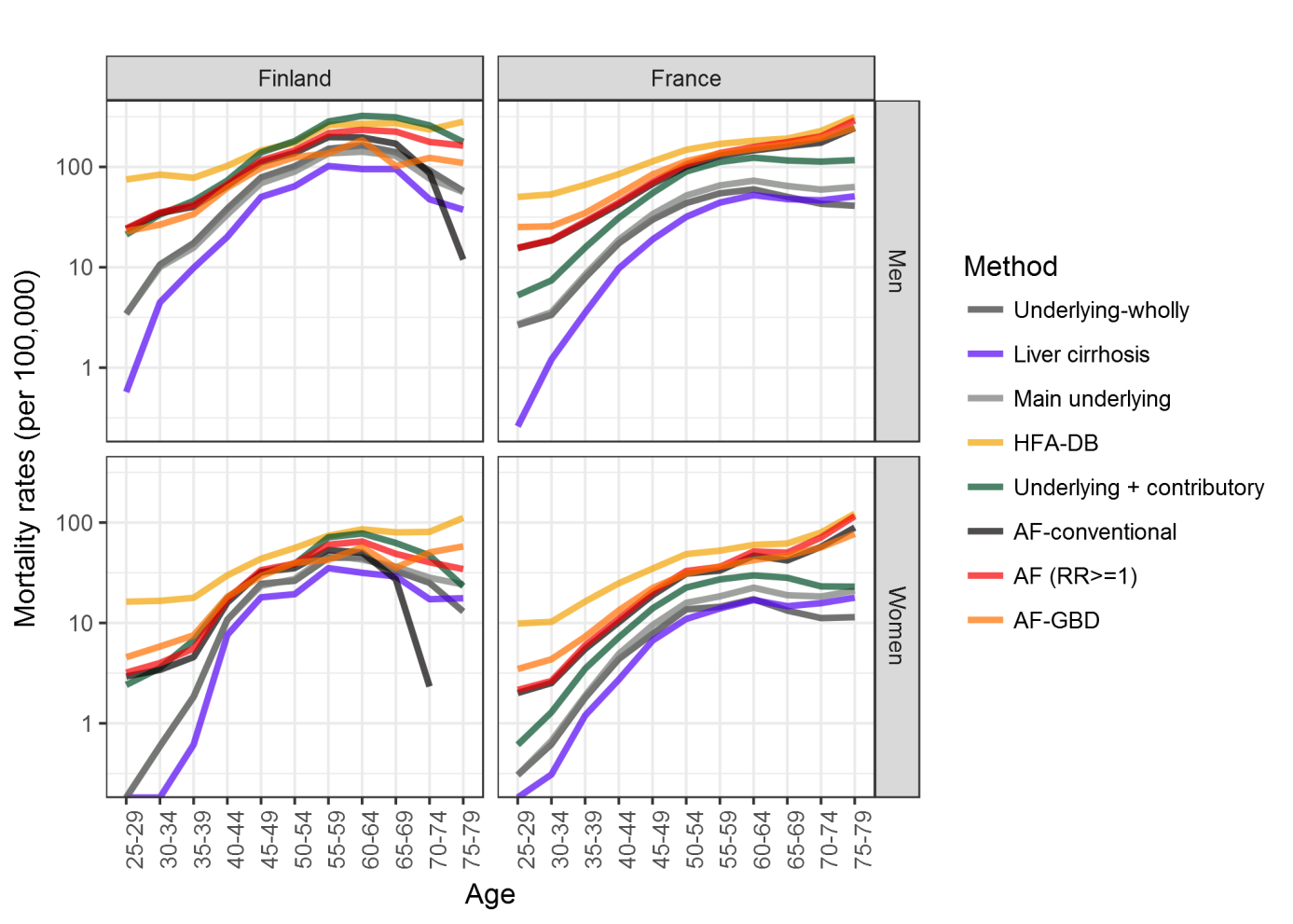


*AF method for Finnish women aged 75-79 is excluded from the plot for visualization reasons as the rate is negative (-35.4).
